# Spatial Distribution Dataset of All Constructive Grass Species in Tibetan Grasslands under 2024 and 2060

**DOI:** 10.1038/s41597-025-06277-x

**Published:** 2026-01-30

**Authors:** Guoyong Tang, Qingwan Li, Shunbin Wang, Jinkai Gu, Qinglin Li, Shengjian Xiang, Wanchi Li

**Affiliations:** 1 Chuxiong Normal University, Chuxiong, China; 2 Institute of Highland Forest Science, Chinese Academy of Forestry Sciences, Kunming, China; 3 Yuanmou Desert Ecosystem Research Station, National Long-Term Scientific Research Base of comprehensive control, Chuxiong, Yunnan China; 4 Institute of International Rivers and Eco-security, Yunnan University, Kunming, China

**Keywords:** Grassland ecology, Environmental health, Phenology

## Abstract

The Tibetan grassland is the principal ecosystem in a high-altitude, ecologically fragile region, providing vital services for biodiversity and local livelihoods. Constructive grass species play a pivotal role in maintaining the structure and function of grassland ecosystems. In this study, we constructed a comprehensive dataset of the spatial distribution of all constructive grass species (44) in Tibetan grasslands under current (2024) and four future (2060) climate scenarios by leveraging species distribution models and high-resolution ecological data. Our dataset encompasses each individual constructive grass species habitat distribution, including expansion, contraction, and stability. This dataset will be applied to climate-change impact assessment on individual species, grassland-type shift projection, and multi-stressor ecosystem-dynamics evaluation. It is essential for supporting grassland management, biodiversity conservation, and livestock production in high-altitude grassland ecosystems.

## Background

Tibetan grasslands cover more than 60% of the Tibetan Autonomous Region, and they constitute a crucial component of the Qinghai-Tibet Plateau ecosystem^1^. Tibetan grasslands play an irreplaceable role in mitigating global climate change and maintaining biodiversity, serving as a crucial ecological foundation in this high-altitude, fragile environment^2^. They also function as the primary resource for pastoral production, sustaining local livelihoods and shaping socio-economic well-being in Tibetan communities^3^.

In grassland ecology, constructive species—also known as “建群种” in Chinese vegetation ecology literature -are the key taxa that define and distinguish vegetation types in grassland ecosystems^4,5^. Unlike simply abundant or companion species, constructive species shape the overall structure and function of plant communities by regulating habitat conditions, mediating ecosystem processes, and serving as diagnostic indicators of grassland integrity^6,7^. They are therefore indispensable in vegetation classification and in understanding ecological dynamics^8,9^. In Tibetan grasslands, 44 constructive species collectively represent all 17 grassland types, capturing diverse functional traits and ecological niches^10–12^. Such coverage makes them particularly suitable for modeling species distributions and assessing the impacts of climate change on grassland ecosystems.

Species Distribution Models (SDMs) have become essential tools for predicting the spatial distribution of species^13,14^. Commonly applied approaches include Maximum Entropy (MaxEnt)^15–17^, Generalized Linear Models (GLMs)^18,19^, and Random Forests^20,21^, all of which are based on the principle that species distributions are determined by their ecological requirements and environmental tolerances. Among these, MaxEnt has shown outstanding performance, as it can model distributions using presence-only data and performs reliably even with limited occurrence records^22,23^. Previous studies in Tibetan grasslands have mainly focused on individual species or specific grassland types^24–26^, revealing that temperature and precipitation are major factors influencing species distributions, as well as predicting potential elevation shifts and expansions of suitable habitats under future climate scenarios. However, no comprehensive dataset currently integrates the spatial distributions of all constructive grass species across the entire region. To address this gap, we employed MaxEnt as the primary modeling tool and integrated multi-dimensional ecological variables to project the distributions of 44 constructive grass species representing all 17 grassland types. The resulting dataset provides a unified, high-resolution resource for understanding grassland dynamics and assessing ecological responses to future climate change.

## Methods

### Study area

Tibet, located in southwestern China, spans 78°25′ to 99°06′E and 26°44′ to 36°32′N, with an average elevation above 4,000 m. The region experiences significant climatic variability, with annual temperatures ranging from −2.8 °C to 11.9 °C and precipitation from 360 mm to 550 mm, decreasing from the southeast to the northwest^27^. These gradients strongly influence vegetation distribution and the structure of grassland ecosystems.

### Constructive grass species of Tibetan grassland

In this study, we examined 44 constructive grass species that collectively represent all such species identified across Tibetan grasslands. These species were determined based on findings from the Second Grassland Survey of the Tibet Autonomous Region. Species occurrence data (1970–2024) were acquired from multiple sources: (1) The Global Biodiversity Information Facility (GBIF, http://www.gbif.org); (2) The National Specimen Resources Sharing Platform (http://www.naii.org.cn); (3) The Chinese Digital Herbarium (http://www.cvh.ac.cn).

To ensure data quality, we used Enmtools to filter, deduplicate, and clean the occurrence records. Specifically, records were processed as follows: (1) Deduplication and Spatial Filtering: We retained only one valid occurrence per 1 km × 1 km grid cell to reduce spatial autocorrelation. (2) Outlier Detection and Removal: Geographically inconsistent records were identified and removed based on species range constraints, ensuring that occurrence points aligned with known species distributions. (3) Final Data Export: After screening, the cleaned occurrence records, including latitude and longitude coordinates, were saved in CSV format for subsequent modeling.

Detailed species information, including family, genus, representative grassland type, and the number of occurrence records for each constructive grass species, is provided in Table 1.

**Table 1:** Constructive grass species of Tibetan grassland.

Grass Species	Code	Family	Genus	Grassland Type	Distribution Points
*Ajania fruticulosa*	AF	Asteraceae	*Ajania*	Temperate steppe-desert, Temperate desert	23
*Argentina anserina*	AA	Rosaceae	*Argentina*	Azonal lowland meadow	67
*Artemisia minor*	AM	Asteraceae	*Artemisia*	Alpine grassland	70
*Artemisia stracheyi*	AS	Asteraceae	*Artemisia*	Alpine grassland	61
*Artemisia vestita*	AV	Asteraceae	*Artemisia*	Temperate grassland	47
*Artemisia wellbyi*	AW	Asteraceae	*Artemisia*	Temperate grassland, Alpine grassland	77
*Artemisia younghusbandii*	AY	Asteraceae	*Artemisia*	Temperate grassland, Alpine grassland	22
*Bistorta macrophylla*	BM	Polygonaceae	*Bistorta*	Montane meadow	102
*Bistorta vivipara*	BV	Polygonaceae	*Bistorta*	Montane meadow	25
*Caragana versicolor*	CV	Fabaceae	*Caragana*	Temperate desert grassland, Alpine grassland, Alpine desert grassland	50
*Carex alatauensis*	CA	Cyperaceae	*Carex*	Montane meadow, Alpine meadow	54
*Carex cercostachys*	CCE	Cyperaceae	*Carex*	Alpine meadow	36
*Carex deasyi*	CD	Cyperaceae	*Carex*	Alpine meadow	56
*Carex littledalei*	CL	Cyperaceae	*Carex*	Alpine meadow	53
*Carex moorcroftii*	CM	Cyperaceae	*Carex*	Alpine meadow, Alpine grassland, Alpine desert grassland	67
*Carex neesii*	CN	Cyperaceae	*Carex*	Alpine meadow	18
*Carex parvula*	CP	Cyperaceae	*Carex*	Alpine meadow, Alpine meadow-steppe	144
*Carex tibetikobresia*	CT	Cyperaceae	*Carex*	Alpine meadow	21
*Christolea crassifolia*	CCR	Brassicaceae	*Christolea*	Alpine desert	39
*Dasiphora fruticosa*	DF	Rosaceae	*Dasiphora*	Alpine grassland	90
*Dasiphora parvifolia*	DP	Rosaceae	*Dasiphora*	Alpine grassland	58
*Elymus nutans*	EN	Poaceae	*Elymus*	Montane meadow	86
*Juniperus pingii*	JP	Cupressaceae	*Juniperus*	Alpine meadow-steppe	46
*Krascheninnikovia ceratoides*	KCE	Amaranthaceae	*Krascheninnikovia*	Alpine desert grassland, Temperate desert, Temperate steppe-desert	50
*Krascheninnikovia compacta*	KCO	Amaranthaceae	*Krascheninnikovia*	Alpine desert grassland, Alpine desert	16
*Leymus secalinus*	LS	Poaceae	*Leymus*	Alpine meadow	74
*Myrtama elegans*	ME	Tamaricaceae	*Myrtama*	Azonal lowland meadow	13
*Orinus thoroldii*	OT	Poaceae	*Orinus*	Alpine grassland, Temperate steppe-desert	67
*Pennisetum flaccidum*	PF	Poaceae	*Pennisetum*	Temperate grassland	57
*Poa alpina*	PA	Poaceae	*Poa*	Alpine meadow	14
*Poa litwinowiana*	PL	Poaceae	*Poa*	Montane meadow	38
*Puccinellia himalaica*	PH	Poaceae	*Puccinellia*	Alpine meadow	27
*Sophora davidii*	SD	Fabaceae	*Sophora*	Warm-temperate shrub tussock	16
*Sophora moorcroftiana*	SM	Fabaceae	*Sophora*	Temperate grassland	30
*Spiraea canescens*	SPC	Rosaceae	*Spiraea*	Temperate grassland, Montane meadow	43
*Stevenia canescens*	STC	Brassicaceae	*Stevenia*	Alpine desert, Montane meadow	30
*Stipa bungeana*	SB	Poaceae	*Stipa*	Temperate grassland	18
*Stipa capillacea*	SC1	Poaceae	*Stipa*	Temperate meadow-steppe, Alpine meadow-steppe	34
*Stipa caucasica*	SC2	Poaceae	*Stipa*	Temperate desert grassland, Temperate steppe-desert, Alpine desert grassland	43
*Stipa purpurea*	SP	Poaceae	*Stipa*	Alpine grassland, Alpine meadow-steppe	230
*Stipa roborowskyi*	SR	Poaceae	*Stipa*	Alpine grassland	52
*Stipa subsessiliflora*	SS	Poaceae	*Stipa*	Alpine grassland	32
*Suaeda corniculata*	SUC	Amaranthaceae	*Suaeda*	Alpine meadow	24
*Triglochin palustris*	TP	Juncaginaceae	*Triglochin*	Marsh type rangeland	40

### Ecological variables

MaxEnt (Version 3.4.4) was used to predict the spatial distribution of 44 constructive grass species based on observed occurrences and ecological variables (Table 2) under current (2024) and future (2060) conditions. These factors are key drivers shaping vegetation distribution and offer a solid basis for assessing grassland dynamics under changing environmental conditions^28^. Current and projected climate data (2024–2060) were obtained from WorldClim (http://www.worldclim.org). The current climate conditions (2024) in this study were represented using historical climate data from 2020–2021 provided by WorldClim. Future climate projections were derived from the CMIP6 BCC-CSM2-MR climate model, covering the periods 2021–2040 and 2040–2060.

**Table 2:** Data sources and environmental factors used for model parameterization.

Date Source	Dataset	Description and unit	Available at
Climate	bio1*	Annual Mean Temperature (°C)	http://www.worldclim.org
	bio2*	Mean Diurnal Range (°C)	
	bio3*	Isothermally	
	bio4*	Temperature Seasonality (*)	
	bio5*	Max Temperature of Warmest Month (°C)	
	bio6*	Min Temperature of Coldest Month (°C)	
	bio7*	Annual Temperature Range (°C)	
	bio8*	Mean Temperature of Wettest Quarter (°C)	
	bio9*	Mean Temperature of Driest Quarter (°C)	
	bio10*	Mean Temperature of Warmest Quarter (°C)	
	bio11*	Mean Temperature of Coldest Quarter (*) (°C)	
	bio12*	Annual Precipitation (*) (mm)	
	bio13*	Precipitation of Wettest Month (mm)	
	bio14*	Precipitation of Driest Month (mm)	
	bio15*	Precipitation Seasonality (Coefficient of Variation)	
	bio16*	Precipitation of Wettest Quarter (mm)	
	bio17*	Precipitation of Driest Quarter (mm)	
	bio18*	Precipitation of Warmest Quarter (mm)	
	bio19*	Precipitation of Coldest Quarter (mm)	
Soil factors	t-esp*	Exchangeable sodium percentage (%)	http://www.fao.org/soil-portal
	t-ece*	Soil electrical conductivity (dS/m)	
	t-clay*	Clay content (%wt)	
	t-cec-soil*	Cation exchange capacity (cmol/kg)	
	t-cec-clay*	Soil cation exchange content (cmol/kg)	
	t-CaSO4*	Soil sulfate content (%weight)	
	t-CaCO3*	Soil carbonate content (%weight)	
	t-bs*	Base saturation (%)	
	t-gravel*	Soil gravel content (%vol.)	
	t-oc*	Soil organic carbon content (%weight)	
	t-ph-H2O*	Soil pH (-log(H+))	
	t-ref-bulk*	Soil bulk density (kg/dm?)	
	t-sand*	Sand content (%wt.)	
	t-silt*	Silt content (%wt.)	
	t-teb*	Soil exchangeable base (coml/kg)	
	t-tusda-tex-clay*	Soil texture classification (name)	
Topographic factors	aspect*	Aspect (°)	https://www.gscloud.cn
	dem	Elevation (m)	
	slope*	Slope (°)	
Drought factors	ai*	Aridity index (%)	http://data.tpdc.ac.cn
Other factors	ndvi*	Normalized difference vegetation index	http://www.resdc.cn
	hfp*	Human footprint	http://ciesin.org
Tibet autonomous region	—	—	https://www.gscloud.cn

Environmental factors marked with an asterisk (*) have a spatial resolution of 1000 m, while those without an asterisk have a resolution of 30 m; Fields prefixed with “t-” represent upper soil properties (0–30 cm).

Although Tibetan grasslands consist of distinct types, including alpine meadows, steppes, and other grassland types, we did not include current grassland type as an explicit environmental variable in our models. This decision was based on the following considerations: (1) The primary aim of this study is to assess climate-driven changes in species distribution, rather than the influence of pre-existing vegetation types. Since climate change can drive shifts in grassland composition, using static grassland type data as a predictor may not fully capture these dynamics. (2) Key environmental factors such as temperature, precipitation, and soil properties inherently shape grassland types, making them indirect yet sufficient predictors in our models. Future studies integrating dynamic vegetation models could further refine predictions by incorporating land cover transitions and ecological succession dynamics.

The selection of 2060 as the future projection year is based on the Chinese carbon neutrality goal, which aims for significant policy and environmental transitions by mid-century. Beyond 2060, grassland management policies and their long-term impacts remain uncertain, making it challenging to incorporate post-2060 projections into this study. Therefore, our modeling focuses on  projected changes within the 2024-2060 timeframe.

Future climate scenarios were derived from the CMIP6 BCC-CSM2-MR model under four Shared Socioeconomic Pathways (SSPs), representing distinct greenhouse gas emission trajectories and socio-economic developments. These scenarios allow us to assess how varying levels of anthropogenic influence might shape the distribution of grassland species. Four greenhouse gas emission pathways were evaluated under the CMIP6 model^29^:

**SSP126** (sustainable development, +2 °C warming)

This scenario represents a low-emission pathway where global greenhouse gas emissions are significantly reduced through strong climate policies, widespread adoption of renewable energy, and sustainable land-use practices. It provides insights into the best-case scenario for grassland conservation and climate adaptation.

**SSP245** (moderate development, +3 °C warming)

This scenario assumes a middle-ground approach with moderate emission reductions and continued economic growth. It reflects a balanced trade-off between development and climate mitigation, making it a likely real-world scenario in the absence of aggressive policy interventions.

**SSP370** (regional development, +4.1 °C warming)

This scenario envisions a fragmented world with limited international cooperation, leading to continued reliance on fossil fuels and regional disparities in climate action. It represents a high-risk future where grassland degradation could accelerate due to unregulated land use and extreme climate conditions.

**SSP585** (conventional development, +5 °C warming)

This scenario assumes a continued increase in greenhouse gas emissions due to an economy heavily reliant on fossil fuels. It represents the worst-case scenario, where global temperatures rise by approximately 5 °C by the end of the century, leading to severe ecological consequences such as desertification and biodiversity loss.

By considering these four SSP scenarios, this study aims to provide a comprehensive assessment of potential changes in grassland species distribution under different climate futures. The results will help guide adaptive management strategies and inform conservation planning efforts for the Tibetan grasslands.

Detailed data information is provided in Table 2.

In this study, only climate variables (temperature- and precipitation-related factors) were updated according to the four SSP scenarios, while non-climatic variables such as soil, vegetation, and human footprint were held constant at their 2024 levels due to the absence of reliable future projections. This assumption is consistent with common SDM practices and allows the analysis to isolate the effects of climate change on species distributions. While human footprint was included as a static variable to represent anthropogenic influence, our modeling primarily focused on climate-driven distributional shifts. Future work could further disentangle the relative contributions of human disturbance and climate change by incorporating dynamic land-use or socio-economic projections.

### Data processing and modeling workflow

#### Variable selection and input preparation

For each species, we first ran a preliminary MaxEnt model using all available environmental variables and recorded their contribution rates. Variables with a contribution rate of zero were excluded. Next, we performed a pairwise Spearman correlation analysis on the remaining variables using SPSS 27.0 to identify highly correlated pairs (absolute correlation coefficient |r| ≥ 0.8)^30^. Spearman correlation was chosen because it measures monotonic relationships and is more suitable for handling non-normally distributed and non-linear ecological data. In each correlated pair, we retained only the variable with the higher contribution rate and removed the other to minimize multicollinearity. Finally, we used the selected variables to run the final MaxEnt model for each species. This stepwise selection process was conducted individually for each species to ensure that the environmental variables used in modeling accurately reflected species-specific responses to ecological gradients. Consequently, the final set of environmental variables varied according to each species’ ecological preferences.

#### Model setup

The data were randomly split into training (75%) and testing (25%) subsets, repeated ten times to ensure robustness^31^. Default MaxEnt parameters were used unless otherwise specified. Model outputs were generated in ASCII format and converted to raster maps (“ASC to Raster” in ArcGIS 10.8) for detailed visualization of each species habitat suitability. The final habitat suitability results represent the average output across the ten replicate runs, ensuring consistency and reliability.

#### Suitability reclassification

To estimate total potential suitable habitat, we reclassified each species’ suitability map into four categories^32^: (1) Non-suitable (0 ≤ P < 0.2); (2) Low suitability (0.2 ≤ P < 0.4); (3) Medium suitability (0.4 ≤ P < 0.6); (4) High suitability (0.6 ≤ P ≤ 1).

We then defined the sum of low, medium, and high suitability areas as the total potential suitable habitat for each constructive grass species.

## Data Records

The dataset is available at Figshare^33^ and contains spatial distribution maps for 44 constructive grass species in Tibetan grasslands, modeled under current (2024) and four future (2060) climate scenarios. Each species is organized in an individual folder containing the following GeoTIFF files: (1) 2024.tiff: Current climate distribution; (2) 2060_SSP126.tif: Projected distribution under 2 °C warming scenario (sustainable development); (3) 2060_SSP245.tif: Projected distribution under 3 °C warming scenario (moderate development); (4) 2060_SSP370.tif: Projected distribution under 4.1 °C warming scenario (regional development); (5) 2060_SSP585.tif: Projected distribution under 5 °C warming scenario (conventional development).

Each folder, named after the Latin name of the corresponding constructive grass species, is archived in Figshare. The dataset is publicly available under 10.6084/m9.figshare.30290848.

Each GeoTIFF file adheres to the following specifications: (1) Spatial Resolution: 1 km × 1 km; (2) Coordinate System: GCS_WGS_1984.

Pixel values: (1) 1: Indicates suitable habitat (0.2 ≤ P ≤ 1); (2) 0: Represents non-distribution areas.

Cell Size: 0.008333 degrees (~1 km).

## Technical Validation

### Model performance evaluation

**Fig. 1:**
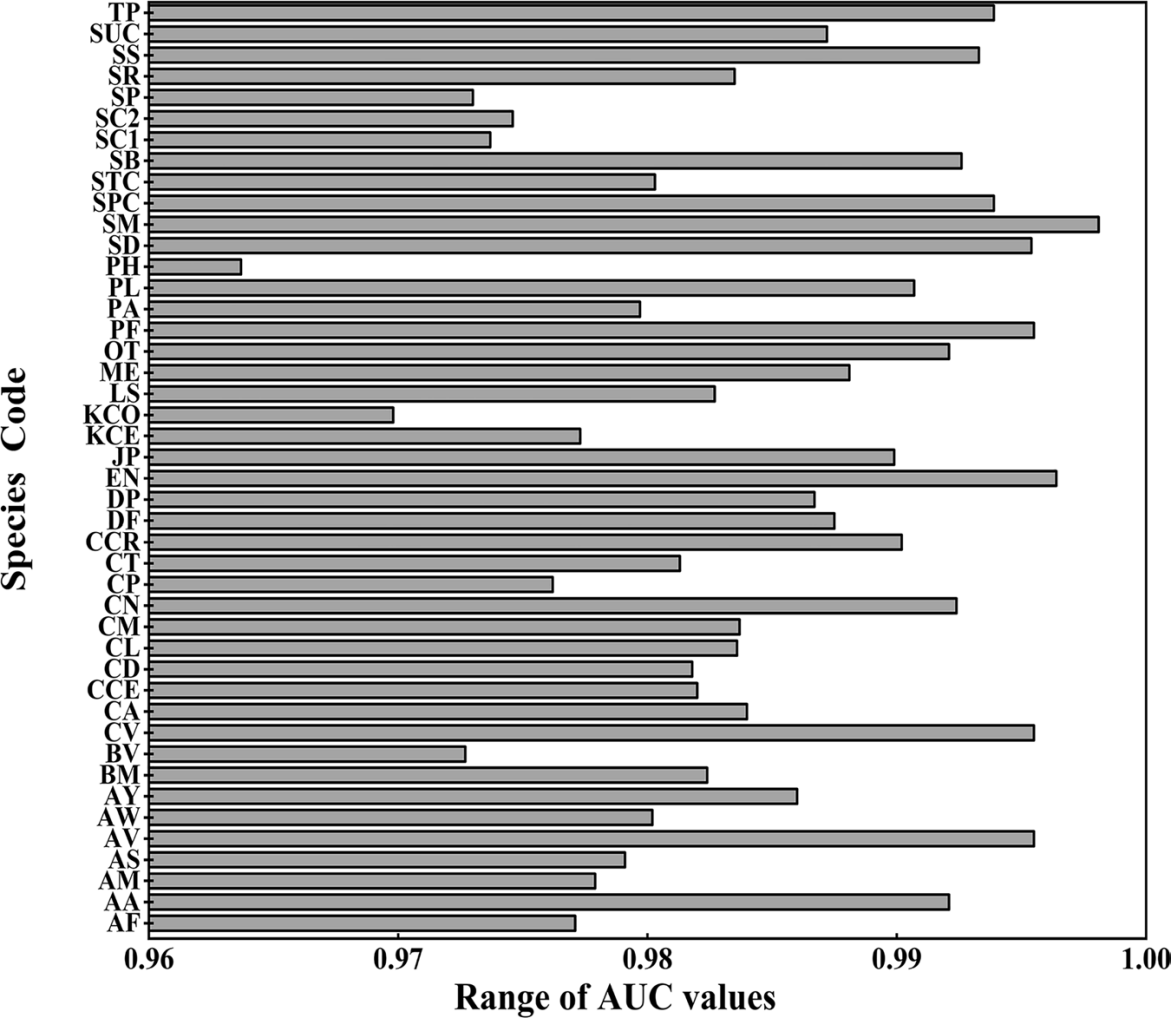
Range of AUC values for constructive grass species. Note: Species codes are listed in Table 1.

Model performance was evaluated using AUC (Area Under the Curve) values, which range from 0.5 (random prediction) to 1.0 (perfect prediction). Models achieving AUC values above 0.8 demonstrated strong predictive reliability, while those ≤ 0.6 were considered to have poor accuracy^34^. In this study, we used AUC to assess the predictive performance of the species distribution models. Under current climate conditions, all species achieved Test AUC values greater than 0.9 (Fig. 1), indicating that the models exhibited strong predictive capability. The difference between Training AUC and Test AUC was small (all less than 0.05), suggesting that the models performed similarly on both training and test datasets without significant overfitting. Therefore, Test AUC can be considered a reliable indicator of model predictive performance. Furthermore, under different future climate scenarios, all Test AUC values remained above 0.9, demonstrating that the models maintained high predictive accuracy across varying climate conditions. This suggests that the models possess good generalization ability and stability, ensuring reliable predictions of species distributions under future climate change.

### Minimizing overfitting

Overfitting was addressed by employing an iterative variable selection process, tailored individually for each species^35^. Spearman correlation analysis was utilized to reduce redundancy among environmental variables, effectively minimizing multicollinearity. This process not only improved model interpretability and stability but also enhanced predictive performance. After variable selection, the AUC values for most specie models demonstrated consistent improvements, with an average increase of 0.02 to 0.05 compared to initial runs without variable filtering.

### Raster data verification

Reclassified raster maps were validated against species occurrence points in ArcGIS 10.8 to ensure alignment with ecological gradients. This process confirmed that the modeled species distributions accurately reflected the expected spatial patterns based on input occurrence data.

## Usage Notes

This dataset offers a robust foundation for ecological research, conservation planning, and sustainable management of Tibetan grasslands. Its applications are diverse and include the following:

### Evaluating the impact of climate change on each individual specie

The dataset provides spatial distribution data for 44 constructive grass species under current and future climate scenarios, enabling precise assessments of climate change impacts on individual species. Researchers can identify species at risk of habitat loss and regions with potential range expansions. These insights are critical for conservation prioritization and resource allocation to mitigate biodiversity loss.

**Fig. 2:**
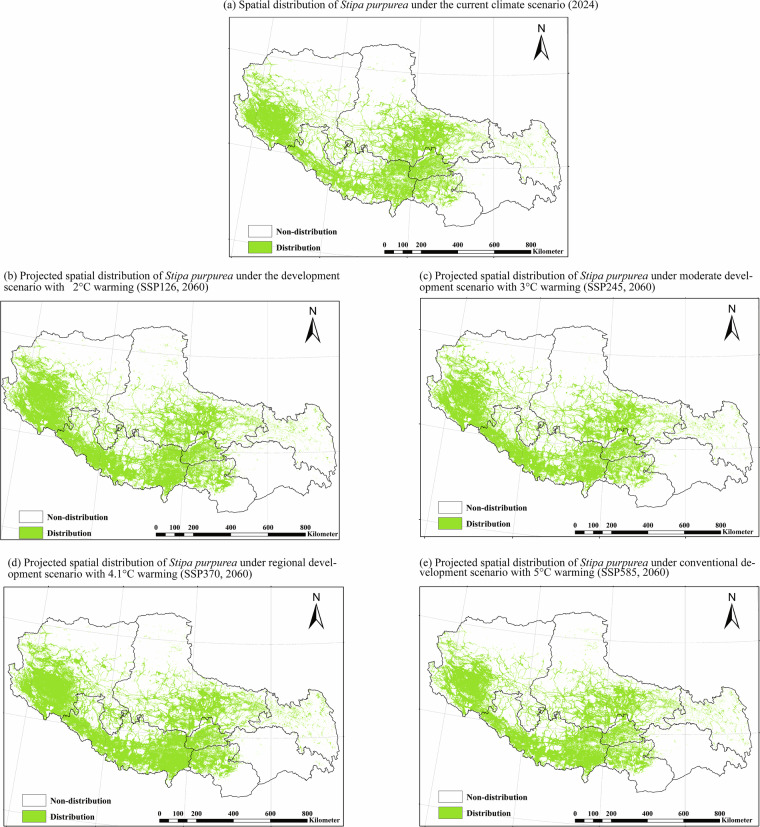
Spatial distribution of *Stipa purpurea* under 2024 and future climate (2060).

**Fig. 3:**
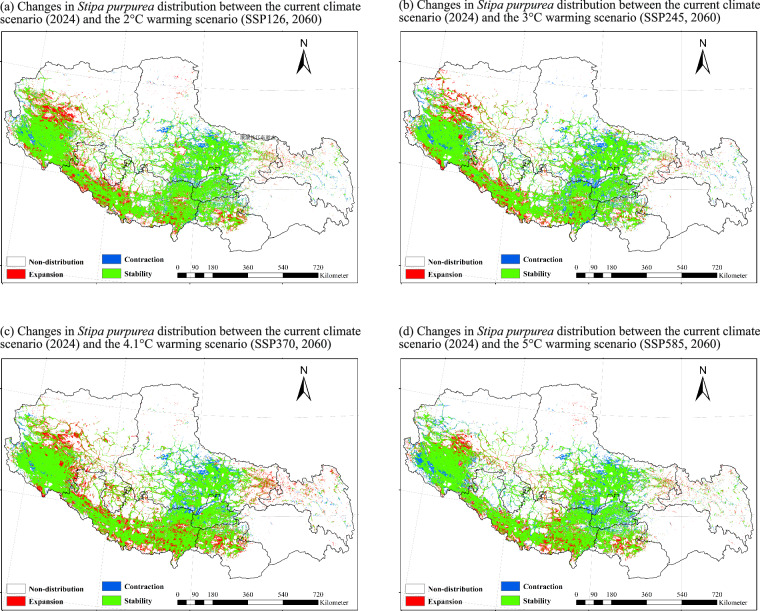
Expansion and contraction areas of *Stipa purpurea* from 2024 to 2060.

To demonstrate the dataset’s capacity, Stipa purpurea was selected as a representative species (Figs. 2, 3) due to its ecological significance in maintaining Tibetan grassland stability^36^.

1. Fig. 2 illustrates the spatial distribution of S. purpurea under 2024 and 2060 climate scenarios, highlighting potential shifts in suitable habitat across Tibetan grasslands.
2. Fig. 3 provides an example of how users can further analyze the dataset to identify areas of species expansion, contraction, and stability across different climate scenarios. This figure does not represent pre-processed results within the dataset but serves as a demonstration of potential applications for ecological research and conservation planning.

These figures serve as illustrative examples of how the dataset can be utilized for analyzing climate change impacts on individual species. They are not intended to provide comprehensive results or predictive conclusions but rather to demonstrate the dataset’s potential applications.

### Assessing the future distribution on each of grassland types

Grassland types—ranging from alpine meadows to steppes and desert grasslands—are defined by distinct assemblages of constructive species. By aggregating these species’ distributions, the dataset facilitates analysis of grassland type shifts across spatial and temporal scales under varying climate scenarios. This information is critical for land-use planning, including identifying areas suitable for restoration or sustainable grazing, and for understanding transitions between grassland types essential for ecological connectivity.

### Projecting future grassland changes

By integrating climate projections and species distribution data, this dataset allows for modeling the potential dynamics of Tibetan grasslands under diverse climate scenarios and anthropogenic pressures. Researchers can simulate shifts in grassland extent, composition, and ecological functions over time, identify regions at risk of degradation or desertification, and pinpoint areas suitable for targeted restoration. Additionally, tracking changes in key species provides early warning signals of grassland transformations, guiding proactive management and policy interventions.

### Supporting biodiversity conservation

This dataset provides high-resolution distribution data for 44 constructive grass species, offering a valuable resource for identifying priority areas for conservation and habitat restoration. By highlighting regions vulnerable to habitat loss or suitable for range expansion, it supports the development of climate adaptation strategies. Furthermore, the dataset fosters collaboration, enabling comparative studies with grassland systems worldwide and advancing global biodiversity conservation efforts.

### Facilitating comparative studies across ecosystems

With high-resolution maps, the dataset enables researchers to compare Tibetan grasslands with other ecosystems, identifying shared patterns and unique responses to climate. These comparisons support the development of generalized strategies for global grassland management and contribute to meta-analyses aimed at understanding universal ecological drivers and patterns.

### Limitations

Although the models generally exhibited high predictive accuracy, a few species (e.g., *Poa alpina*) had relatively sparse occurrence records, which may introduce uncertainty into their projected distributions. However, these species are naturally rare and often occur in specific alpine microhabitats, meaning their ecological importance may be better represented when analyzed together with co-occurring constructive species. Users are therefore advised to interpret the results of these rare species with caution and to combine them with field validation or complementary datasets where possible.

Additionally, while human footprint was included as a static variable to represent anthropogenic influence, this study primarily focused on climate-driven distributional shifts. Future work could incorporate dynamic land-use or socio-economic data to better separate the effects of human disturbance and climate change.

## Data Availability

All species distribution maps and metadata generated in this study have been deposited in Figshare and are publicly available at 10.6084/m9.figshare.30290848.

## Code availability

No specific programs or codes were used in this study.
